# Intimate partner violence and maternal educational practice

**DOI:** 10.1590/S1518-8787.2017051006848

**Published:** 2017-03-29

**Authors:** Josianne Maria Mattos da Silva, Marília de Carvalho Lima, Ana Bernarda Ludermir

**Affiliations:** I Programa de Pós-Graduação em Saúde da Criança e do Adolescente. Universidade Federal de Pernambuco. Recife, PE, Brasil; II Departamento Materno Infantil. Centro de Ciências da Saúde. Universidade Federal de Pernambuco. Recife, PE, Brasil; III Departamento de Medicina Social. Centro de Ciências da Saúde. Universidade Federal de Pernambuco. Recife, PE, Brasil

**Keywords:** Violence Against Women, Spouse Abuse, Domestic Violence, Child Abuse, Child Rearing, Family Relations, Cross-Sectional Studies

## Abstract

**OBJECTIVE:**

The objective of this study is to analyze the association between intimate partner violence against women and maternal educational practice directed to children at the beginning of formal education.

**METHODS:**

This is a cross-sectional study, carried out between 2013 and 2014, with 631 mother/child pairs, registered in the Family Health Strategy of the Health District II of the city of Recife, State of Pernambuco, Brazil. It integrates a prospective cohort study designed to investigate the consequences of exposure to intimate partner violence in relation to the child who was born between 2005 and 2006. The maternal educational practice has been assessed by the Parent-Child Conflict Tactics Scale and the intimate partner violence by a questionnaire adapted from the Multi-Country Study on Women’s Health and Domestic Violence of the World Health Organization. Intimate partner violence referred to the last 12 months and was defined by specific acts of psychological, physical, and sexual violence inflicted to women by the partner. The crude and adjusted prevalence ratios were estimated for the association studied, using log-binomial regression.

**RESULTS:**

The prevalence of intimate partner violence was 24.4%, and violent maternal educational practice was 93.8%. The use of non-violent discipline was mentioned by 97.6% of the women, coexisting with violent strategies of discipline. Children whose mothers reported intimate partner violence presented a higher chance of suffering psychological aggression (PR = 2.2; 95%CI 1.0–4.7).

**CONCLUSIONS:**

The violence suffered by the mother interferes in the parental education. The findings show high prevalence of violent maternal educational practice, pointing to the need for interventions that minimize the damage of violence in women and children.

## RESUMO

**OBJETIVO:**

Analisar a associação entre a violência pelo parceiro íntimo contra a mulher e a prática educativa materna direcionada às crianças no início da escolaridade formal.

**MÉTODOS:**

Estudo transversal, realizado entre 2013 e 2014, com 631 pares mãe/criança, cadastradas na Estratégia de Saúde da Família do Distrito Sanitário II da cidade do Recife, Pernambuco. Integra o estudo de coorte prospectivo delineado para investigar as consequências da exposição à violência pelo parceiro íntimo para a criança que nasceu da gestação que ocorreu entre 2005 e 2006. A prática educativa materna foi avaliada pela escala de conflitos *Parent-Child Conflict Tactics Scale* e a violência pelo parceiro íntimo por um questionário adaptado do Estudo Multipaíses sobre a Saúde da Mulher e Violência Doméstica da Organização Mundial da Saúde. A violência pelo parceiro íntimo referiu-se aos últimos 12 meses e foi definida por atos concretos de violência psicológica, física e sexual infligidos à mulher pelo parceiro. Foram estimadas as razões de prevalência brutas e ajustadas para a associação estudada, utilizando-se a regressão log-binominal.

**RESULTADOS:**

A prevalência da violência pelo parceiro íntimo foi de 24,4%, e da prática educativa materna violenta de 93,8%. O uso de disciplina não violenta foi referido por 97,6% das mulheres, coexistindo com estratégias violentas de disciplinamento. As crianças cujas mães relataram violência pelo parceiro íntimo apresentaram maior chance de sofrer agressão psicológica (RP = 2,2; IC95% 1,0–4,7).

**CONCLUSÕES:**

A violência sofrida pela mãe interfere na educação parental. Os achados demonstram alta prevalência de prática educativa materna que perpassa pela violência, o que aponta para a necessidade de intervenções que minimizem os prejuízos da violência na mulher e na criança.

## INTRODUCTION

Intimate partner violence (IPV) is a high-frequency phenomenon observed in the Multi-Country Study on Women’s Health and Domestic Violence, conducted by the World Health Organization with 24,097 women aged between 15 and 49 years, of which 15% to 71% reported physical or sexual violence by intimate partner at some point in their lives^10^. The Brazilian sample consisted of 940 women from the city of São Paulo and 1,188 women from the Zona da Mata of Pernambuco, showing the prevalence of 9.3% and 14.8%, respectively, for the occurrence of physical or sexual violence by intimate partner in the last 12 months^25^.

A previous study^27^ has examined the association between IPV with a risk of child abuse. Among the results, 40% of the women had suffered violence by the current partner and were twice at risk of perpetrating abuse against their children compared to women with no history of IPV. High levels of maternal stress were associated with the perpetration of the four types of violence analyzed (physical aggression, spanking, psychological aggression, and negligence).

Research studies indicate that the presence of IPV increases the risks of physical punishment^1^ and child abuse by the mother (physical punishment, negligence, psychological aggression, and sexual violence)^2^ ^,^ ^9^ ^,^ ^12^ ^,^ ^30^.

To analyze the association between the mother who has experienced violence and the use of physical punishment as educational practice, a study in Peru^9^ has found that being a victim of IPV increases the risk of the mother physically punishing their children. The research of Chang et al.^2^ has indicated an association between psychological violence from the partner and child abuse. In the investigation of disciplinary practices used by mothers who are victims of violence, Kelleher et al.^12^ have observed that any type of violence by the partner was associated with higher frequencies of physical and psychological aggression and negligence of the mother towards the child, thus concluding that the violence suffered by the women is associated with a more aggressive parenting.

The IPV places the woman in a hostile, stressful, and debilitating environment, affecting her relationship with her children^23^ ^,^ ^24^. The development of post-traumatic symptoms can generate feeling of insecurity in this bond^16^, influencing the parental behavior of the woman^14^ ^,^ ^23^. She can develop compensatory behaviors, giving the children a proper parenting and safeguarding them from the reality of violence, or, on the other hand, she can develop aggressive behaviors, reflecting the experience of violence in the lives of the children^20^. As a result, the IPV can raise the risks of using improper disciplinary methods, such as corporal punishment by the mother^20^.

However, there are few studies on the association of IPV against woman with the violence practiced by the mother against her child, especially in the Brazilian reality. Thus, the objective of this study has been to investigate the association between intimate partner violence (IPV) against women, in the last 12 months, and maternal educational practice (MEP) directed to their children at the beginning of formal education.

## METHODS

This is a cross-sectional study performed in the Health District II of Recife, State of Pernambuco, Brazil, between July 2013 and December 2014, with women and children participating in the third phase of a prospective cohort study designed to investigate the consequences of intimate partner violence against women during pregnancy, postpartum, and the last seven years in relation to the mental health of the women and the psychosocial development of the children born between July 2005 and October 2006. The Health District II is predominantly a residential area, focused on the medium and low income segment. It has five special areas of social interest (ZEIS), where approximately 60% of the population lives, being one of the districts that has the highest proportion of inhabitants in ZEIS in the city^17^.

The cohort study has conducted three phases of data collection. The first two took place in 2005 and 2006. In the first phase, all pregnant women (n = 1,133), aged 18 to 49 years old, with 31 weeks or more of gestation, registered in the Family Health Strategy (FHS) of Health District II of Recife were considered as eligible. Contacts with the pregnant women who had no prenatal care in the Family Health Unit (FHU) and with those who had no regular prenatal care were made at their homes. These pregnant women were identified from the records of community health agents and included in the study.

Of the 1,133 eligible women, 1,121 (98.9%) were interviewed and, of these, 1,057 were re-interviewed in the postpartum period (second phase). For this research, the third phase of the cohort, 644 women (61.5%) who participated in the second phase were interviewed. Between the second and the third phase, five women died, 391 were not found because of a change of address, and 17 refused to remain in the research. Among the children, four died. Two children who were given to other families, two children who lived with other family members, and five pairs of twins were excluded from the study with their respective mothers. The population of the study consisted of 631 mother-child pairs.

The data were collected by higher education professionals, trained and experienced in research on the health of woman and child or violence. In the training, ethical issues and the need for collecting accurate information were emphasized. We conducted simulated interviews, in addition to a pilot study in the Health District VI of Recife. The interviews were mostly conducted in the homes of the participants, privately, or at another place convenient for the women. There was no direct interview with the children and the information was referred to by their mother.

The MEP was assessed by the Parent-Child Conflict Tactics Scale, adapted and validated for Brazil by Reichenheim and Moraes^22^. The scale contains twenty-two items, which investigates domestic violence in the parental educational practice from response to situations that occur in everyday life. In filling the scale, the respondent must report the occurrence of some behaviors adopted in relation to the child. The scale assesses the educational practice in three dimensions: non-violent discipline (disciplinary practices as an alternative to corporal punishment), psychological aggression (verbal and symbolic acts whose intention is to cause psychological pain or fear), and physical aggression (corporal and physical punishments). The physical punishment dimension is subdivided into corporal punishment, minor physical abuse, and serious physical abuse. In this study, the two types of physical abuse were grouped in the scale of physical aggression.

For the analysis of the Parent-Child Conflict Tactics Scale, we considered as a positive case of violent MEP the women’s answer “yes” to at least one of the items in the scale of psychological aggression or physical aggression. In this way, the frequency of (physical and psychological) violence corresponds to the confirmation of at least one act of violence against the child in a 12-month recall period. The analysis of the non-violent discipline occurred similarly, assessing the occurrence or not of a certain behavior, considering as positive the affirmation of at least one of the items of the respective scale.

The IPV issues had as reference the questionnaire of the Multi-Country Study on Women’s Health and Domestic Violence, conducted by the World Health Organization. We have defined as intimate partner the boyfriend, partner, or ex-partner with whom the women kept sexual-affective relationships, regardless of formal union or cohabitation. Intimate partner violence referred to the last 12 months and was identified by specific acts of psychological, physical, and sexual violence inflicted to women by the partner. Physical violence was characterized as: physical aggression or use of weapons or objects to produce lesions; psychological violence, such as threatening behaviors, humiliations, and insults; and sexual violence, such as sexual intercourse imposed by physical force or threats and imposition of acts that were considered as demeaning. Women who answered “yes” to at least one of the questions that make up each type of violence were considered as positive cases. Additional information on the methods of the study are reported in other publications^17^ ^,^ ^26^.

We also analyzed the following covariates of the children: age (6-8, > 8 years), gender (male, female), education (≤ 2nd grade; ≥ 3rd grade), and if they went to day care before school (yes, no); and of the mothers: age (≤ 27 years, ≥ 28 years), race (white, non-white), with partner (no, yes), years of study (between 0 and 4, ≥ 5), and monthly income (more than one minimum wage [equivalent to R$678 at the time of the study], less than one minimum wage or no income).

The data were entered into the program EpiInfo, version 3.5.3 for Windows, with double entry of data by different typists. Subsequently, the application Validate was used to check for typos and then we cleaned and checked the consistency of the data. For the statistical analysis we used the program SPSS, version 15 for Windows. We estimated the prevalence of IPV (physical, psychological, and sexual) that occurred exclusively or superimposed, in addition to the prevalence of MEP in the non-violent discipline and in the physical and psychological dimension. Initially, the bivariate analysis was performed to identify the presence of potential associations between the covariates studied and exposure – IPV – and outcome – MEP. The association between IPV and MEP was estimated by the crude and adjusted ratio of prevalence. The statistical significance was assessed by the Chi-square test, considering a 95% confidence interval and the value of p < 0.05. Log-binomial regression was used to analyze the independence of the association between IPV and MEP. The covariates included in the model were those described in the literature as potential confounding factors and which, in this study, were associated with MEP and IPV with the value of p < 0.10.

The research has met the ethical requirements imposed by Resolution 196/96, of the Brazilian Health Council. The study has been approved by the Research Ethics Committee with Human Beings of the *Universidade Federal de Pernambuco* (Notice 194,672). All participants have signed the informed consent.

## RESULTS

As a result of the longitudinal nature of the original cohort study, 408 of the women interviewed in the postpartum period were not re-interviewed in this study (Table 1). However, the comparison between them showed no statistically significant differences in relation to IPV and the demographic and socioeconomic variables.

**Table 1:** Comparison of the socioeconomic and demographic characteristics and intimate partner violence of the women who participated (N = 644) and did not participate (N = 408) of the third phase. Recife, State of Pernambuco, Brazil, 2013-2014.

Variable	Participants (n = 644)		Non-participants (n = 408)		p
	
	n	%	n	%	
Age (years)					
≥ 28	432	67.1	313	76.7	0.13
≤ 27	212	32.9	95	23.3	
Race*					
White	139	21.7	71	17.4	0.09
Non-white	502	78.3	337	82.6	
With partner					
No	95	14.7	44	10.8	0.06
Yes	549	82.2	364	89.2	
Years of study*					
0-4	139	21.7	97	23.8	0.51
≥ 5	502	79.3	311	76.2	
IPV					
No	501	77.8	311	76.2	0.55
Yes	143	23.2	97	23.7	

IPV: prevalence physical, psychological, and sexual

* Missing data for three participants.

In relation to maternal educational practice, 91.4% of the interviewees reported using at least one act of psychological aggression, while 82.4% reported at least one act of physical aggression. The use of non-violent discipline was mentioned by 97.6% of the women as educational strategy, coexisting with violent strategies of discipline. In the analysis of the MEP, we considered as violent MEP the cases of physical or psychological aggression. The prevalence of violent PEM was 93.8% (Table 2).

**Table 2:** Prevalence of types of intimate partner violence against women, in the last 12 months, and maternal educational practice. Recife, State of Pernambuco, Brazil, 2013-2014.

Variable	n	%
Intimate partner violence against women		
Psychological violence	74	11.7
Physical and psychological violence	45	7.1
Exclusive physical or psychological violence associated with sexual violence	15	2.4
Physical, psychological, and sexual violence	20	3.2
Total cases of violence	154	24.4
Maternal educational practice with the child		
Non-violent discipline	616	97.6
Physical aggression	520	82.4
Psychological aggression	577	91.4
Total cases of violence	592	93.8

Regarding the prevalence of IPV, 24.4% of the women reported having experienced at least one of the types of violence in the last year, being the psychological violence the most predominant (11.7%). The IPV was subsequently grouped into yes and no (Table 3). The distribution of the socioeconomic and demographic characteristics of the mothers and children indicated that most of the women under study were aged 28 years or more (86.7%), were non-white (82.2%), and lived with a partner (80.7%). Women with five or more years of study (83.9%) and with no income or income of less than one minimum wage (66.4%) were also predominant. Regarding the characteristics of the children, more than half were aged between six and eight years (52.9%) and were females (50.9%), and most did not go to day care before school (88.4%) and were in third grade, or more, of elementary school (76.9%).

**Table 3:** Distribution of the socioeconomic and demographic characteristics of the women and demographic characteristics of the children and their association with intimate partner violence against women. Recife, State of Pernambuco, Brazil, 2013-2014.

Variable	N = 631		Intimate partner violence						
	
	n	%	No	%	Yes	%	PR	95%CI	p
Mother									

Age (years)									
≥ 28	547	86.7	414	86.8	133	86.4	1.00		
≤ 27	84	13.3	63	13.2	21	13.6	1.01	0.9–1.2	0.89
Race^a^									
White	112	17.8	88	18.5	24	15.7	1.00		
Non-White	516	82.2	387	81.5	129	84.3	1.05	0.9–1.2	0.42
With partner									
No	122	19.3	96	20.1	26	16.9	1.00		
Yes	509	80.7	381	79.9	128	83.1	1.05	1.0–1.2	0.38
Years of study^b^									
≥ 5	527	83.9	406	85.5	121	79.1	1.00		
0-4	101	16.1	69	14.5	32	20.9	1.13	1.0–1.3	0.06
Monthly income									
≥ 1 minimum wage	212	33.6	163	34.2	49	31.8	1.00		
< 1 minimum wage/No income	419	66.4	314	65.8	105	68.2	1.03	0.9–1.1	0.59

Child									

Age (years)									
> 8	297	47.1	241	50.5	56	36.4	1.00		
6-8	334	52.9	236	49.5	98	63.6	1.15	1.1–1.3	0.002
Gender									
Male	310	49.1	235	49.3	75	48.7	1.00		
Female	321	50.9	242	50.7	79	51.3	1.01	0.9–1.1	0.90
Day care									
No	558	88.4	429	89.9	129	83.8	1.00		
Yes	73	11.6	48	10.1	25	16.2	1.17	1.0–1.4	0.04
Education level^c^									
≤ 2nd grade	145	23.2	109	23.0	36	23.5	1.00		
≥ 3rd grade	482	76.9	365	77.0	117	76.5	1.00	0.9–1.1	0.89

Missing values: ^(a)^ 3, ^(b)^ 3, ^(c)^ 4.

In the bivariate analysis of the socioeconomic and demographic characteristics of the women and the demographic characteristics of the children with IPV (Table 3), women who had children aged between six and eight years and who went to day care showed statistically significant association with IPV. Women with less education were the ones who most reported having suffered IPV (Table 3).

The bivariate analysis of MEP with the socioeconomic and demographic characteristics of the women and the demographic characteristics of the children (Table 4) showed that women with less education more physically abused their children, and younger children (6 to 8 years) were more physically abused (PR = 1.42; 95%CI 1.0–2.0; p = 0.04).

**Table 4:** Association of the socioeconomic and demographic characteristics of the woman and demographic characteristics of the child with maternal educational practice. Recife, State of Pernambuco, Brazil, 2013-2014.

Variable	Maternal educational practice											

	Non-violent discipline				Physical aggression				Psychological aggression			
		
	No (%)	Yes (%)	PR (95%CI)	p	No (%)	Yes (%)	PR (95%CI)	p	No (%)	Yes (%)	PR (95%CI)	p
Mother												

Age												
≥ 28	15 (2.7)	532 (97.3)	1.00		94 (17.2)	453 (82.8)	1.00		48 (8.8)	499 (91.2)	1.00	
≤ 27	0 (0)	84 (100)	0.00^a^	0.24^b^	17 (20.2)	67 (79.8)	0.85 (0.5–1.4)	0.49	6 (7.1)	78 (92.9)	1.23 (0.5–2.8)	0.62
Race^c^												
White	1 (0.9)	111 (99.1)	1.00		25 (22.9)	87 (77.7)	1.00		9 (8.0)	103 (92.0)	1.00	
Non-white	13 (2.5)	503 (97.5)	0.35 (0.1–2.7)	0.49^b^	84 (16.3)	432 (83.7)	1.37 (0.9–2.0)	0.13	44 (8.5)	472 (91.5)	0.94 (0.5–1.9)	0.87
With partner												
No	3 (2.5)	119 (97.5)	1.00		18 (14.7)	104 (85.2)	1.00		7 (5.7)	115 (94.3)	1.00	
Yes	12 (2.4)	497 (97.6)	1.04 (0.3–3.6)	1.00^b^	93 (18.3)	416 (81.7)	0.81 (0.5–1.3)	0.36	47 (9.2)	462 (90.8)	0.62 (0.3–1.3)	0.22
Years of study												
≥ 5	10 (1.9)	517 (98.1)	1.00		100 (19.0)	427 (81.0)	1.00		46 (8.7)	481 (91.3)	1.00	
0-4	5 (4.9)	96 (95.1)	0.38 (0.1–1.1)	0.08^b^	11 (10.9)	90 (89.1)	1.74 (1.0–3.1)	0.05	8 (7.9)	93 (92.1)	1.10 (0.5–2.3)	0.79
Monthly income												
≥ 1 minimum wage	4 (1.9)	208 (98.1)	1.00		44 (20.7)	168 (79.2)	1.00		18 (8.5)	194 (91.5)	1.00	
< 1 minimum wage/No income	11 (2.6)	408 (97.4)	0.72 (0.2–2.2)	0.57	67 (16.0)	352 (84.0)	1.30 (0.9–1.8)	0.14	36 (8.6)	383 (91.4)	0.99 (0.6–1.7)	0.97

Child												

Age (years)												
> 8	7 (2.4)	290 (97.6)	1.00		62 (20.9)	235 (79.1)	1.00		30 (10.1)	267 (89.9)	1.00	
6-8	8 (2.4)	326 (97.6)	0.98 (0.4–2.7)	0.97	49 (14.7)	285 (85.3)	1.42 (1.0–2.0)	0.04	24 (7.2)	310 (92.8)	1.41 (0.8–2.4)	0.19
Gender												
Male	6 (1.9)	304 (98.1)	1.00		48 (15.5)	262 (84.5)	1.00		28 (9.0)	282 (91.0)	1.00	
Female	9 (2.8)	312 (97.2)	0.69 (0.3–1.9)	0.47	63 (19.6)	258 (80.4)	0.79 (0.6–1.1)	0.17	26 (8.1)	295 (91.9)	1.12 (0.7–1.9)	0.68
Day care												
No	13 (2.3)	545 (97.7)	1.00		100 (17.9)	458 (82.1)	1.00		47 (8.4)	511 (91.6)	1.00	
Yes	2 (2.7)	71 (97.3)	0.85 (0.2–3.7)	0.69^b^	11 (15.1)	62 (84.9)	1.19 (0.7–2.1)	0.55	7 (9.6)	66 (90.4)	0.88 (0.4–1.9)	0.74
Education level												
≤ 2nd grade	1 (0.7)	144 (99.3)	1.00		26 (17.9)	119 (82.1)	1.00		12 (8.3)	133 (91.7)	1.00	
≥ 3rd grade	14 (2.9)	468 (97.1)	0.24 (0.0–1.8)	0.21^b^	83 (17.2)	399 (82.8)	1.04 (0.7–1.6)	0.84	40 (8.3)	442 (91.7)	1.00 (0.5–1.9)	0.99

^a^ Prevalence ratio and 95%CI impossible to be calculated.

^b^ Fisher’s exact test.

^c^ One value missing.

In the analysis of the association between IPV and MEP, the results indicate that children whose mothers reported having suffered IPV are twice as likely to suffer psychological aggression (p = 0.04) (Table 5).

**Table 5:** Association of intimate partner violence, in the last 12 months, with maternal educational practice. Recife, State of Pernambuco, Brazil, 2013-2014.

Variable	Maternal educational practice											

	Non-violent discipline				Physical aggression				Psychological aggression			
		
	No	Yes	Crude PR	Adjusted PR^a^	No	Yes	Crude PR	Adjusted PR^b^	No	Yes	Crude PR	Adjusted PR^c^
											
	n (%)	n (%)	(95%CI)	(95%CI)%	n (%)	n (%)	(95%CI)	(95%CI)	n (%)	n (%)	(95%CI)	(95%CI)
Intimate partner violence												
No	9 (1.9)	468 (98.1)	1.00	1.00	92 (19.3)	385 (80.7)	1.00	1.00	47 (9.8)	430 (90.1)	1.00	1.00
Yes	6 (3.9)	148 (96.1)	0.48 (0.2–1.3)	0.51 (0.2–1.5)	19 (12.3)	135 (87.7)	1.56 (1.0–2.5)	1.50 (0.9–2.5)	7 (4.5)	147 (95.4)	2.17 (1.0–4.7)	2.17 (1.0–4.7)
p			0.22	0.21			0.05	0.10			0.04	0.04

^a^ Adjusted for years of study of the woman.

^b^ Adjusted for the age of the child and years of study of the woman.

^c^ No adjustment carried out, as no covariate met the criteria established for entry in the model (p < 0.10).

## DISCUSSION

In this study, we have found a prevalence of 24.4% of women victims of IPV in the last 12 months. Among the types of violence, the psychological one was the most prevalent, with 11.7%. Exclusive physical or psychological violence associated with sexual violence had the lowest prevalence, 2,4% Garcia-Moreno et al.^10^, in the Multi-Country Study, have found prevalence of physical and sexual violence, in the last 12 months, of 8.3% and 2.8% in the city of São Paulo and 12.9% and 5.6% in the Zona da Mata of Pernambuco, respectively. Schraiber et al.^25^, analyzing data from this same study, have found prevalence of psychological violence, in the last year, of 18.7% and 24.2% for São Paulo and Pernambuco, respectively. In the previous phase of the cohort study to which this article is linked^26^, IPV is estimated in 22.6% in the puerperium period. By type of violence, the prevalence was: 12.1%, 19.3%, and 3.7%, for physical, psychological, and sexual violence, respectively.

In studies on violence, the prevalence of IPV is influenced by several factors, such as the cultural and socioeconomic ones^10^ ^,^ ^25^ ^,^ ^26^ and it expresses the importance of gender issues for its understanding^7^. Violence between intimate partners involves the historical construction of power relations that mark the masculine and feminine characteristics, configuring a hierarchical relationship between man and woman^3^. In this context, the IPV also expresses a gender inequality.

Among the types of violence, a higher frequency of psychological violence is a common finding in the studies mentioned^10^ ^,^ ^25^ ^,^ ^26^. In our results, exclusive physical violence was less frequent, being present together with psychological violence or accompanied by sexual violence. In turn, sexual violence was not found in isolation, but always superimposed on other types of violence (physical or psychological). Schraiber et al.^25^ consider that sexual violence usually accompanied by physical violence is a common occurrence. However, sexual violence is also present in cases when the woman feels coerced, having sexual intercourse because of her fear of the attitude of the partner in relation to her refusal.

The frequency of IPV observed showed value similar to previous studies^25^ ^,^ ^26^. It is important to note that a underreporting of events can occur in studies on violence because, as this is a delicate and sensitive issue, it may cause embarrassment or fear about the reported information. Remembering the violence suffered can be a trying experience, causing fear and shame, which can make women feel unavailable to talk about the subject. It is admitted^25^ that, while women would hardly report episodes of violence that did not occur – given the condition of shame, guilt, and stigma –, on the other hand, for the same reasons, she could frequently hide the facts.

Another result of this study shows that, even with the possibility of omission in relation to MEP regarding the school-age child, there was a high prevalence of violent MEP (93.8%), both in the physical aggression dimension (82.4%) and in the psychological aggression dimension (91.4%). In Minas Gerais, a high percentage of psychological violence (95.6%) and physical violence (94.4%) was also found^21^.

Violent child discipline appears in other important research studies. The study of Vitolo et al.^28^ has shown a frequency of 43.3% for physical punishment; spanking was the most frequent attitude, mentioned by 36.1% of the interviewees. The authors have observed that parents/educators who held the belief that spanking was something educational had higher chances of physically hitting their children compared with those who believed that punishment was unnecessary, with frequencies of 64.8% and 42.5%, respectively (p = 0.002; OR = 2.5; 95%CI 1.4–4.5).

Corporal punishment was reported by 88.1% of the participants of a research that interviewed children and adolescents^29^. The researchers point out that the passage from punishment to abuse is very tenuous and the abuse may be a result of both the lack of knowledge of other educational strategies and the harm that the coercive educational practices can cause in the child.

The ignorance on the issues related to child development can be associated with the reproduction of punitive and educational practices, which is combined with the traditional understanding of education that associates punishment with education, leading to the reproduction of educational models learned from the family and culture, making it difficult to change the educational behavior towards appropriate educational practices^19^. This study draws attention to the occurrence of violence naturalized in the daily attitudes of the parental figures. Women who had violent MEP also used non-violent discipline (97.6%). Sani^24^ has also found a similar result; when comparing the educational practices of women victims and non-victims of violence to the practices considered as appropriate (such as giving advice and explaining to the children what they did wrong), they did not differ in the two groups; on the other hand, abuse and punishments differed, showing higher frequency among victimized women.

In this research, we have found that exposure to IPV interfered with the maternal behavior in relation to the child, increasing the risk of the use of violent educational practices. The relationship between IPV and a more violent maternal parenting is consistent with other research studies^1^ ^,^ ^27^. In a study that has analyzed the behavior of women who suffered partner violence, Levendosky and Graham-Bermann^13^ have noted that violence was a significant predictor in maternal parenting, concluding that physical and psychological abuse interfered with the parenting, being the psychological abuse the most damaging one, relating to antisocial behavior in children.

The literature^15^ ^,^ ^24^ ^,^ ^27^ discusses the implications of IPV in maternal parenting. It can negatively affect the psychological functioning of women, generating higher stress levels than in women who are not victims of violence^14^ ^,^ ^15^. Its traumatic effect may influence the parental behavior and the ability to care^14^, increasing the chance of developing depression, abusing the children, and being negligent^27^. It is associated with the inability to respond to the needs of the child with tenderness and affection, and the increase in hostility and disconnection, reflecting the dysregulated affection of the mothers^16^. Thus, the normalizing experience of violence can be repeated in the mother-child relationship^24^.

Children are very vulnerable to the responses and the affection of the mother, and when she is a victim of violence, she can develop physical symptoms^18^, in addition to being able to present a depressive disorder^4^ ^,^ ^6^ ^,^ ^17^ and post-traumatic stress symptoms^8^ ^,^ ^15^. The parenting of those women can be compromised and expressed by inappropriate responses to the needs of the child.

Regarding the use of disciplinary methods, Sani^23^ points out that the use of coercive strategies to handle children can be a way to minimize or avoid more severe acts of the partner on the child.

As educational practice, physical punishment and corporal punishment are ineffective and even damage the children as they produce negative consequences for their development. The use of corporal punishment, since this is usually accompanied by a speech of the parents that they love the child and what they are doing is for their sake, can lead to an association between pain and love, teaching them to use the same method in other life situations, or even make them support aversive situations that should be ended^29^. In addition, these practices can cause in the child negative feelings such as hostility, fear and anxiety, and the development of aggressive^16^ and antisocial behavior^5^ ^,^ ^11^. Psychological violence, in turn, can produce feelings of guilt, shame, anger, social isolation, psychosomatic aspects, phobias, repetition of nightmares, impairment of mental health, among other effects in the child^7^.

Because of the loss of the cohort, our study had some limitations. However, to minimize the effect of the losses and the underestimation of the violence, we adopted measures such as the selection of female interviewers trained to address the subject according to ethical principles appropriate to the subject. The reduction in the number of participants, in relation to the previous period of data collection, could have influenced the estimates of IPV. However, when we compare the women interviewed in the second phase of the cohort study with those who did not participate in this study, we did not find any statistically significant difference in relation to IPV and the demographic and socioeconomic variables.

This study, while presenting limitations, represents a research whose theme is still little investigated: the relationship between IPV and other types of violence. It contributes to the literature by addressing the problem in two parts: the woman victim of partner violence and the child abused by the mother, exposing both the violence that is expressed in gender inequality and power relations, and the one that is naturalized in parental relationships, being reproduced in the educational practices.

The research indicates that violent acts may occur together, in which the person abused can also be an aggressor and violent educational practices, while being disseminated, are disguised as socially permissible disciplinary practices.

The results point to the need to offer clarification to women on maternal care, by professionals in the Family Health Strategy, advising them about the repercussions of this care in the child, and the risks that the context of violence provides for the physical and mental health of both the woman and child.
